# ‘A basic understanding’; evaluation of a blended training programme for healthcare providers in hospital-based palliative care to improve communication with patients with limited health literacy

**DOI:** 10.1186/s12909-022-03685-0

**Published:** 2022-08-11

**Authors:** Janneke Noordman, Ruud Roodbeen, Leonie Gach, Lotte Schulze, Jany Rademakers, Maria van den Muijsenbergh, Gudule Boland, Sandra van Dulmen

**Affiliations:** 1 Nivel (Netherlands Institute for Health Services Research), PO Box 1568, 3500 BN Utrecht, the Netherlands; 2 Research Department, Breuer & Intraval, Research and Consultancy, Groningen, The Netherlands; 3 Department of Family Medicine, Care and Public Health Research Institute (CAPHRI), Maastricht University, Maastricht, The Netherlands; 4 Pharos, Dutch Centre of Expertise on Health Disparities, Utrecht, The Netherlands; 5 Department of Primary and Community Care, Radboud University Medical Center, Radboud Institute for Health Sciences, Nijmegen, The Netherlands; 6 Faculty of Caring Science, Work Life and Social Welfare, University of Borås, Borås, Sweden

**Keywords:** Education, Blended training, Evaluation, Healthcare providers, Communication, Shared decision-making, Patients, Limited health literacy, Palliative care

## Abstract

**Background:**

The non-curative setting makes communication and shared decision-making in palliative care extremely demanding. This is even more so for patients with limited health literacy. So far, research in palliative care focusing on shared decision-making with patients with limited health literacy is lacking. Recent research from our team indicates that the assessment of these patients’ understanding of their situation and the implementation of shared decision-making in palliative care, needs improvement.

**Methods:**

To improve communication and decision-making, especially with patients with limited health literacy, we developed and evaluated a blended training programme for healthcare providers. The training programme comprised of an e-learning and a team training. The evaluation was performed by 1. conducting interviews (*n* = 15) focused on evaluating the whole programme and, 2. coding video-recorded outpatient consultations on the extent to which providers involved patients in decision-making before (*n* = 19) and after (*n* = 20) the intervention, using the 5-item OPTION coding instrument.

**Results:**

The interviews showed that healthcare providers valued the skills they had learned during the e-learning and team training. Providers specifically valued the teach-back technique, learned to use simpler wording and felt better able to recognize patients with limited health literacy. Many providers reported a change in communication behaviour as a consequence of the training programme. Suggestions for improvement for both e-learning and training were, amongst others, a follow-up team training course and a new scenarios for the e-learning about discussing palliative care. For both the pre- and the post-measurement, involving patients in decision-making lies between a minimal and a moderate effort; differences were not significant.

**Conclusions:**

The e-learning and team training were valued positively by the healthcare providers. Adaptations to the e-learning have been made after evaluation. The e-learning has been implemented in several hospitals and medical education. To improve shared decision-making in practice a more sustained effort is needed.

**Supplementary Information:**

The online version contains supplementary material available at 10.1186/s12909-022-03685-0.

## Background

Each year, around 56.8 million people worldwide need palliative care [1]. Palliative care is concerned with relieving serious health-related suffering for people with a severe illness [1]. Improving patients’ quality of life and addressing their needs and those of significant others are important aims of palliative care [1]. The recommended approach for choosing the most appropriate, patient-centered care path is shared decision-making (SDM) [2]. SDM is a process in which healthcare provider (HCP) and patient together decide which medical policy is best for the patient, taking into account all options, advantages and disadvantages, patient preferences and circumstances [3]. In the Netherlands, HCPs are legally required to fully inform their patients about every available option and related risks and consequences, to take notice of patients’ situation and personal needs, and to invite patients to ask questions [4].

In palliative care, actively participating in SDM can be demanding for patients as a result of their emotional and psychological vulnerability, reduced cognitive abilities and the prospect of death [5, 6]. Additionally, HCPs experience difficulties in involving patients due to time pressure and other organisational circumstances [7, 8]. This is even more challenging for patients with limited health literacy (LHL). People with LHL lack the skills to find, understand and apply information about health and healthcare. LHL hampers communication and SDM with HCPs because it affects the ability to ask questions, to understand information and to reflect and plan ahead [9, 10]. Almost 48% of the European population is considered to have LHL [11], with a prevalence of 25% in the Netherlands [12]. Although LHL is most common among low(er) educated persons, males, elderly (65 years or older) and people who judge their health as poor(er) [11], LHL is also context-dependent and can therefore affect all people.

So far, limited research has been carried out in palliative care focusing on SDM and LHL patients [13]. Recent research from our team indicates that the assessment of LHL patients’ understanding of their situation [14] and the implementation of SDM in palliative care, need improvement [15]. To improve communication and decision-making in hospital-based palliative care, we therefore developed and evaluated a blended training programme for healthcare professionals to improve communication with LHL patients. The training programme comprised of an e-learning and a team training, called ‘Goed Begrepen’ in Dutch (‘A basic understanding’). We made use of well-known learning principles capturing knowledge, attitude and behaviour by means of education, modelling, practicing and rehearsing. The aim of this paper is to examine to what extent this training programme improved HCPs’ communication and decision-making in hospital-based palliative care, especially for LHL patients.

## Method

### Design

A mixed method design was used to evaluate a blended training programme for HCPs working in hospital-based palliative care, consisting of an e-learning and a team training called ‘A basic understanding’. The evaluation was executed by conducting interviews and coding video-recorded outpatient consultations. The interviews with participating HCPs focused on evaluating the whole programme. The extent to which HCPs involved patients in decision-making during their recorded consultations was measured using the 5-item ‘‘Observing Patient Involvement in Decision-Making’’ (OPTION) instrument [16–18], before and after the intervention. Video-recordings of consultations are a valid method for examining communication between healthcare providers and patients [19].

#### The e-learning

The e-learning was developed by Dialogue Trainer (technique) and Pharos, Dutch Centre of Expertise on Health Disparities (content), in collaboration with and based on the outcomes of video-observations and subsequent (reflected practice) interviews carried out by Nivel [7, 14, 20]. For example, quotes from the real-life recorded consultations between HCPs and LHL patients in the palliative phase of their disease were used as input for the scenarios of the e-learning [14]. The goal of the e-learning was to help HCPs in hospital-based care to adapt and improve their communication skills with LHL patients in the palliative phase of their disease. The e-learning includes: 1) virtual scenarios of encounters between HCPs and LHL patients. HCPs can play these scenarios to practice conversations with virtual LHL patients and improve their communication skills with respect to recognizing LHL patients, adapting information provision to LHL patients, using teach-back, and to improve their SDM with LHL patients; 2) information about how to recognize LHL patients, adapt communication and how to use supportive materials, including for instance lists of easy to understand medical words; 3) useful interventions developed by others regarding communication, SDM and palliative care; 4) an observation list for HCPs to observe and analyse their own SDM behaviour; 5) patient education materials in plain language about ‘normal dying’, palliative sedation, euthanasia, spiritual care, reanimation and ventilation in an Intensive Care (IC).

Several experts (e.g., General Practitioners (GPs), medical specialists, SDM experts, palliative care experts, researchers, experience experts) and medical students pilot tested the e-learning and the scenarios in it. The pilot testing consisted of testing the content (for example, is the information medically correct?), procedure (for example, the flow of the conversation; the reactions of the patient) and language (is the provided information understandable?) of the e-learning. The e-learning consist of four parts: 1) recognizing patients with LHL and discuss health literacy with patients; 2) plain and comprehensible communication and teach-back; 3) SDM; 4) a ‘library’ of all videos and tools mentioned or used in the e-learning, supplemented by a number of additional tools. See Additional file 1 for visuals of the e-learning (Figs. 1,2,3, and 4).The duration of the e-learning was 2 h on average. The e-learning is available in Dutch through: https://pharosleerplatform.nl/ (it is called ‘Goed Begrepen’). Accreditation (CME points) for HCPs are included. The e-learning has already been implemented in all participating hospitals and in several academic courses.

#### Team training

An experienced trainer from Pharos delivered a face-to-face training for the HCPs in two of the participating hospitals (duration: 2 to 3 h). HCPs from different disciplines and departments within one hospital attended. As a result of COVID-19 measures, the training for the third hospital was delivered online (2 h). The fourth hospital did not participate in the team training of Pharos, because of overlap with another training they already had about SDM (although it did not include health literacy).

The team training focused on health literacy and used palliative case studies. The training aimed to: 1) enhance HCPs’ insight into how LHL patients understand information and advice, and increase their ability to understand how LHL patients raise cues and concerns; 2) teach HCPs to recognize patients with low literacy and limited health literacy skills; 3) teach HCPs how to tailor their communication to the LHL patient; and 4) practice the teach-back technique. The training was very experiential (i.e. based on cases provided by the participating HCPs) while making use of role-plays with a training actor (i.e. the training actor played a patient with LHL). After the role play, the HCPs and actor discussed the good parts of the conversation and the parts that could be improved. Feelings of the actor (as a patient) and experiences of the HCPs were also discussed.

### Respondents

All respondents were HCPs from the four participating hospitals, who care for LHL patients with cancer or COPD in the palliative phase of their disease. The hospitals were located in different regions in the Netherlands (north, east, west and south) and three were university hospitals. All HCPs made use of the e-learning, and HCPs from three hospitals followed the team training.

### Data collection

For the interview study, the coordinator of each participating hospital department sent the contact details of the HCPs to a researcher, who then contacted each individual HCP. Participation was, however, not mandatory. HCPs were interviewed by telephone by one of two researchers (LS or LG) to evaluate the blended training programme. The semi-structured interviews were audio-recorded. All interviews were carried out between July 2020 and January 2021. An initial version of the interview guide was developed by two researchers (JN and LG). Feedback was provided by two other researchers (GB and SvD), after which the interview guide was improved and finalized (see Additional file 2 for interview guide).

The extent to which HCPs involved patients in SDM in their recorded consultations was measured using the 5-item OPTION instrument, before and after the intervention. The OPTION instrument is a reliable and valid method for investigating SDM [16–18]. Five SDM items are coded on a 5-point Likert-type scale, ranging from 0 = ‘‘zero effort observed’’ to 4 = ‘‘exemplary effort’’. Coding of the video-recorded consultations was conducted using BORIS software [21]. The data collection of the recordings, the inclusion criteria and the findings of the application of SDM at pre-measurement (before HCPs followed the training programme) are extensively described in a previous paper [15]. Data collection of the video-recordings in the pre-measurement were carried out between April and October 2018. The procedure for the data collection and inclusion criteria of the post-measurement (i.e. after the programme was completed) was similar to that at pre-measurement [15]. Data collection of the recordings in the post-measurement were carried out between March 2020 and February 2021. In the present paper, a comparison is made between SDM scores at pre- and post-measurement. Hospitals that participated in both the pre- and post-measurement were included for analysis.

### Data analysis

The semi-structured interviews were audio-recorded and transcribed verbatim. Unintelligible parts of the interviews were checked with another researcher. Transcripts were analysed using thematic analysis following the phases described by Braun and Clarke [22]. Transcripts were coded with MAXQDA software [23]. The thematic analysis of 12 interviews was conducted by one researcher (LG) using deductive coding, in which the main themes ‘e-learning’, ‘team training’, ‘communication barriers’ and ‘palliative care’ were decided upon (See interview guide, Additional file 2). To increase reliability, investigator triangulation was applied: two of the interviews were additionally coded by a second researcher (JN). After this initial comparison, the subthemes and elements that emerged during the analysis were discussed among the two researchers, who then came to an agreement. A third researcher (LS) analysed the remaining three interviews, using the developed coding tree. Additional subthemes and elements that were found by the third researcher (LS) were discussed with the second researcher (JN), who then came to an agreement. By analysing subthemes and elements within themes, the researcher who was involved during the entire analysis process (JN) finalized the naming, positioning, and describing of (sub)themes and completed the analyses. Some subthemes or elements that emerged during the thematic analysis are illustrated by quotes, which were translated into English and edited to increase readability without the loss of meaning or context.

For SDM, the total OPTION score is generated by converting the scores to a 0 to 100 scale and then calculating the average, as recommended by the authors of the OPTION [17]. The higher the score, the higher the level of SDM [17]. Differences between the average OPTION scores at pre- and post-measurement were analyzed using a two-sided t-test and *P* < 0.05 was considered statistically significant. All observers were trained by using the OPTION package, that included literature, the OPTION protocol and exercise consultations [17]. In addition, we used four real consultations from this study (two per measurement) to practice with the OPTION. These consultations were not included for the reliability agreement between observers. While coding SDM as part of the pre-measurement [15], the percentage agreement between two observers (RR and JN) was 88%, which indicates a substantial agreement [24]. The percentage agreement between the two observers (LS and LG) of the post-measurement was 65%, indicating moderate agreement. Disagreements between the two observers (LS and LG) were resolved by discussion with a third observer (JN), who also coded the consultations in the pre-measurement. Data was analyzed using the statistical software program STATA [25].

## Results

Seventeen HCPs were interviewed. Data saturation was reached after 15 interviews, after which two more HCPs were interviewed. In the end, interviews from 15 HCPs were included, because two HCPs did not take care of patients with cancer or COPD. The interviews took on average 26 min. Five interviews were conducted with HCPs from the hospital in the west of the Netherlands, four with HCPs from the hospital in the north of the Netherlands, and three interviews with HCPs from hospitals in both the south and east of the Netherlands. Table 1 shows an overview of the background characteristics of the HCPs.

**Table 1:** HCPs’ characteristics (*n* = 15)

	n
**Profession**	
Nurses (specialised)	8
Oncologist/radiologist	4
Pulmonologist	1
Resident	2
**Department**	
Pulmonology (COPD and lung cancer)	7
Radiology	3
Supporting and palliative care	3
Palliative care	2
**Sex**	
Female	12
Male	3

For both the e-learning and the team training three sub-themes emerged and are presented: lessons learned, improvements to the e-learning and the team training and future use. For the theme ‘communication barriers after the training programme’, two sub-themes emerged: barriers in communicating with LHL patients and barriers in communicating about palliative care.

In general, the interviews showed that the HCPs valued the skills they had learned during the e-learning and team training. HCPs specifically valued the teach-back technique and felt better able to recognize LHL patients. Many HCPs reported a change in communication behaviour as a consequence of the e-learning and team training. Some areas of improvement for both e-learning and training were mentioned, such as a follow-up team training course and a new scenarios for the e-learning.

### E-learning

#### Lessons learned

In general, the e-learning was valued by HCPs: *“I thought it was a clear e-learning and pleasant to go through and not very long. (..) I liked the distribution of theory and casuistry [practicing with virtual scenarios].”* (Z3-3). The virtual scenarios were valued by many HCPs: *“What’s also good about it [the virtual scenario’s] is that at a given moment you can only ask a limited number of questions. Which of course is also the case in reality, because there you do not have hours of time for the patient*” (Z1-1). Some HCPs mentioned that they had learned to not overload patients with information: *“I also try to divide a consultation in parts, for example, I will say: we are discussing this now. I will call you back about that […]”.* (Z4-3). Many HCPs mentioned they benefited from the teach-back method: *“it is important to often check whether people have understood my explanation”* (Z2-3). They also learned how to better recognize LHL patients. HCPs mentioned that it is difficult to recognize LHL patients because they are not always the ‘usual suspects’. What surprised HCPs was the high number of LHL patients in the Netherlands (25%). They also mentioned that LHL patients understand information more literally. HCPs learned to use simpler wording or standard sentences from the e-learning. One HCP mentioned that emotional state can influence the extent to which patients understand information, regardless of the degree of education: *“Sometimes you have someone in front of you and even though they are highly educated people, they are stressed or are experiencing emotions or are in a lot of pain and then they do not get everything, or they understand less.”* (Z4-3). Overall, the e-learning was considered a good addition to the team training (or vice versa). Eight HCPs reported to have changed their communication strategies with patients as a result of the e-learning and four felt it might change their communication. Three HCPs indicated that the e-learning had no influence on their communication with patients

#### Improvements

Most cited were technical issues; the e-learning was slow and the sound did not always work. These issues occurred mainly in a network environment and were solved by accessing the e-learning at home. Some HCPs cited that the e-learning was divergent from real life, because they missed the ‘flow’ of the conversation and only had to click for the (right) answer or question: *“You just have to click and that’s not the same as having to really talk and respond to a patient sitting right in front of you”* (Z2-2). Also, some HCPs thought that the e-learning took too much of their time and preferred a ‘quick route’ through the e-learning.

#### Future use

Six HCPs mentioned that they would use the e-learning again and two HCPs were in doubt of future use. Seven of the HCPs cited they would not use the e-learning in the future. They preferred to look up information such as tips and tricks, instead of (partially) repeating the e-learning: *“I don't expect to do it again any time soon. I can imagine that you would, but then it is more that you take out bits and pieces that you think: ‘oh how did you do this again?’. And whether that is exactly this e-learning or whether you look up some general information, it could be from a training or from something else, I think that would be more likely than that you would do the e-learning several times.”* (Z4-2). If the e-learning should be repeated, HCPs would prefer a different version or other scenarios: *“But I don't know if I would want to go through the whole e-learning again. Then I think I would like to have a variant or something. To do exactly the same, I would not find that attractive.”* (Z1-3).

Some HCPs mentioned using other resources, such as the website of Pharos (especially for COPD patients) as well as materials on the teach-back technique.

### Team training

#### Lessons learned

The team training was also considered valuable. Many HCPs mentioned the teach-back technique when asked what they had learned from the team training course. In addition, it was often mentioned that HCPs had learned to use simpler wording: *“I think it really made me aware that we still often say things in a more difficult way than we might think. That we often think that we are doing it in a simple, understandable way, but that we do not do so yet. So, yes, I am much more aware of that. I often really try to keep it simpler than what I already did.”* (Z1-3). Practising with an actor was also perceived as valuable: *“[What I found valuable was] that actress who mimics the behaviour of patients with limited health skills, but in the extreme. Often, it happens in the department that these people may also exhibit the same behaviour, only to a lesser extent, and then it is less likely to be noticed. And because she took it to the extreme, you became aware of certain words that patients say, or behaviour, that those are a sign of ‘Oh I am dealing with a person with limited health [literacy] skills’.”* (Z1-1).

Three HCPs cited that the team training changed their communication with patients and four HCPs doubted whether this was the case.

#### Improvements

Most HCPs preferred the team training in a multidisciplinary setting, but two nurses mentioned that some information in the multidisciplinary training was irrelevant to them. A few HCPs mentioned that the timing of the team training in addition to the e-learning (or vice versa) could be improved: *“There was some time between the training and the e-learning and (..) I think that it is more convenient if there is less time in-between.”* (Z1-1). The HCPs of one hospital participated online in the team training, due to COVID-19 measures. Two of them considered the online training less pleasant and effective than a face-to-face training. Two other HCPs mentioned that the team training resembled other trainings concerning LHL patients. However, they thought it was still useful to repeat this information.

#### Future training

The HCPs preferred repeated training to incorporate what they had learned in the team training in their communication with patients. Some suggested a second training (booster session) after a few weeks or an annual training.

### Communication barriers after the training programme

#### Barriers in communicating with LHL patients

Recognizing the level of health literacy in patients is still a challenge for some HCPs after the training programme, especially since there often is a discrepancy between how people talk and how they understand information. Also, some HCPs mentioned that communication with LHL patients is more difficult as they cannot always explain their problem properly: *“I do notice that some people find it very difficult to express their complaints”* (Z1-3). In addition, HCPs mentioned that information booklets in the hospital are not easily readable for LHL patients. The transfer of information between HCPs is an element that was mentioned in only one interview, but is worth highlighting. An HCP mentioned that it was difficult to notify other HCPs about the LHL level of a patient because there was no specific place in a patient’s medical record to register this and it was perceived to have a negative connotation: *“You don't want to write that in a file. Look with illiteracy […] I think you can still write that down. Because people know that of course […], they probably will not be likely to read that, but that is still a kind of neutral information, that is a fact […]. But the fact that someone has less health literacy skills is a kind of label in my opinion, I would find it difficult to really write that down in their medical file, even though it is really relevant information.”* (Z2-2).

#### Barriers in communicating about palliative care

A lack of knowledge about the existence of the palliative team was mentioned by HCPs as a barrier for patients to get access to the potential benefits of palliative care. HCPs stated that it was not a standard procedure to get the palliative team involved in the palliative phase of disease, except when the patient was hospitalized on the clinical department. HCPs themselves are often not aware of the existence of a palliative team either or do not refer patients to these teams. Some of the HCPs would like to offer a standard intake by the palliative team to every patient in the palliative phase of their disease, so the patient is aware of the palliative team and knows where to find them if they need support. Another barrier for HPCs was still the difficulty in starting the conversation about the approaching end-of-life or visiting the palliative team, as it was considered confrontational for patients: *“What I find difficult is when people are not aware [i.e. patient is not aware of being in the palliative phase of the disease], then I always find it quite difficult to confront them with it. Because on the one hand it can help people to still make important decisions, but a lot of people find that very confrontational to hear and don’t want to talk about it at all. So, I find that difficult”* (Z1-3). Overall, there was no particular manner in which HCPs introduced the topic: *“Sometimes I can be very direct about it if I think the possibility is there, but at other times, when people really cut off any conversation about it, then you cannot always discuss it. It also very much depends on the interaction. So, I don't have one way to discuss that [the approaching end of life].”* (Z4-3).

### Shared decision-making in practice

36 recorded consultations in four hospitals, including 36 patients and 19 HCPs, were coded with the OPTION scale at pre-measurement. At post-measurement, 20 consultations in two hospitals, including 20 patients and 12 HCPs, were coded with the OPTION. The recorded consultations in the two hospitals who participated both in the pre-and post-measurement were compared and analysed; 19 consultations of 10 HCPs at pre-measurement and 20 consultations of 12 HCPs at post-measurement. The departments of the two hospitals were similar in the pre- and post-measurement (i.e. lung disease, radiology or palliative care department). In Table 2 the characteristics of the consultations, HCPs and patients are shown.

**Table 2:** Characteristics of pre-and post-measurement in two hospitals

Pre-measurement	Post-measurement
**Consultations (*n* = 19)**	**Consultations (*n* = 20)**
*Type of consultation*:	*Type of consultation:*
new: 2	new: 1
repeat: 17	repeat: 19
*mean duration*: 22.9 min. (range: 6–69)	*mean duration*: 24.1 min. (range: 8–53)
*Department:*	*Department:*
pulmonology (COPD and lung cancer): 7	pulmonology (COPD and lung cancer): 7
radiology: 8	radiology: 10
palliative care: 4	palliative care: 3
**HCPs (*n* = 10)**	**HCPs (*n* = 12)**
*Type of provider:*	*Type of provider:*
pulmonologist: 1	pulmonologist: 1
oncologist/radiologist: 5	physician assistant pulmonologist: 2
resident: 3	oncologist/radiologist: 5
nurse (specialist): 1	resident: 2
	nurse (specialist): 2
*sex of provider:*	*sex of provider:*
male: 3	male: 4
female: 7	female: 8
**Patients (*n* = 19)**	**Patients (*n* = 20)**
*sex of patient:*	*sex of patient:*
male: 11	male: 14
female: 8	female: 6
*mean age:* 66.9 years (range 48–84)*	*mean age:* 65.1 years (range 52–81)***
education level*:	education level**:
low: 12	low: 10
middle: 5	middle: 3
high: 1	high: 5
*Disease:*	*Disease:*
Cancer: 18	Cancer: 18
COPD: 1	COPD: 2^#^

^*^ 1 missing;** 2 missing;*** 5 missing. ^#^One of these patients was included by the HCP as a COPD patient, but when observing the consultation this patient appeared to have sarcoidosis. Please note that all included patients are patients with LHL skills, based on the screening questions [26, 27] and/or educational level, while taking the expert opinion of the HCP about the patient’s health literacy level into account

### SDM scores before and after the training

At pre-measurement, the mean SDM score (0–100 score) was 38 (SD: 25.3; *n* = 19 consultations). Of the OPTION scale items, the highest average score was observed for Item 1 (score 2.0; HCP drawing attention to or confirming options and the need for a decision), the lowest average scores were observed for Item 2 (score 1.1; HCP reassures or reaffirms support to the patient for becoming informed or deliberate options) and item 4 (score 1.1; HCP makes an effort to elicit the patient’s preferences in response to the options that have been described. When the patient states their preference, the HCP is supportive).

At post-measurement, the mean SDM score (0–100 score) was 41 (SD: 19.5; *n* = 20 consultations). Of the OPTION scale items, the highest average score was observed for Item 3 (score 2.5; HCP gives information or checks understanding about the options that are considered reasonable (this can include taking no action), to support the patient in comparing alternatives), the lowest average scores were observed for item 5 (score 1.2; HCP makes an effort to integrate the patient’s elicited preferences as decisions are made) and Item 2 (score 1.3; HCP reassures or reaffirms support to the patient for becoming informed or deliberate options). See Table 3 for all the OPTION scores at pre- and post-measurement.

**Table 3:** SDM scores before and after the training, using the 5-item OPTION

Items	Pre-measurement	Post-measurement
Mean score	38 (SD: 25.3; *n* = 19 consultations)	41 (SD: 19.5; *n* = 20 consultations)
1. HCP drawing attention to or confirming options and the need for a decision	2.0	1.7
2. HCP reassures or reaffirms support to the patient for becoming informed or deliberate options	1.1	1.3
3. HCP gives information or checks understanding about the options that are considered reasonable (this can include taking no action), to support the patient in comparing alternatives	1.9	2.5
4. HCP makes an effort to elicit the patient’s preferences in response to the options that have been described. When the patient states their preference, the HCP is supportive	1.1	1.5
5. HCP makes an effort to integrate the patient’s elicited preferences as decisions are made	1.4	1.2

Score description: 0 = No effort (zero effort observed in the video-recorded consultation); 1 = Minimal effort (effort to communicate could be implied or interpreted in the video-recorded consultation); 2 = Moderate effort (basic phrases or sentences used in the video-recorded consultation); 3 = Skilled effort (substantive phrases or sentences used in the video-recorded consultation); 4 = Exemplary effort (clear, accurate communication methods used in the video-recorded consultation)

For both the pre- and the post-measurement, this indicates that the extent to which HCPs involve patients in SDM in practice lies between a minimal effort (effort to communicate could be implied or interpreted in the video-recorded consultation) and a moderate effort (basic phrases or sentences used in the video-recorded consultation) [17]. The training programme did not change the SDM level of HCPs (*P* = 0.77; not significant).

## Discussion

This study provides insight into the extent that a blended training programme for HCPs improved their communication and decision-making in hospital-based palliative care with LHL-patients. The evaluation was executed by conducting interviews and coding video-recorded outpatient consultations. The interviews with participating HCPs focused on evaluating the whole programme.

Overall, interviewed HCPs valued the skills they had learned during the blended training programme (e-learning and team training). HCPs specifically valued the teach-back technique, reported to have learned to use simpler wording and felt better able to recognize LHL patients. Many HCPs reported a change in communication behaviour as a consequence of the training programme.

Although HCPs felt better able to recognize LHL patients after the training programme, they still find it difficult to assess a patient’s health literacy level. LHL patients are a very diverse group, including patients with and without lower literacy and although low(er) education is a risk factor for LHL, it is not limited to patients with those education levels [11]. Patients can also become temporally LHL, because of their emotional state in the context of a life-threatening illness and palliative care. Therefore, a distinction could be made between LHL as a trait and LHL as a state [28, 29]. Some HCPs in our study mentioned that communication with LHL patients is more difficult as these patients cannot always explain their problem properly. A previous study also found that HCPs experience difficulty in communicating with LHL patients as patients do not understand HCPs’ explanation, do not react appropriately to their questions or leave the decision about treatment up to them [30]. LHL patients themselves indicated in another study that they often do not understand their diagnosis or problem, while this is a necessary prerequisite to participate in SDM [31]. These findings all suggest that recognizing, supporting and communicating with LHL patients is not easy for HCPs, although these barriers can be lessened by using simple words and sentences, providing not too much information (e.g. limit the information to three essential topics), using teach-back and support the patient emotionally by using affective communication [14]. All these elements are included in our e-learning and team training.

In addition, HCPs mentioned that information booklets are not easily readable for LHL patients. We know that most hospital information is written at B2-level (understandable for 40% of the population), whereas B1 (understandable for 80% of the population), or even better A2-level (understandable for 95% of the population), is recommended. The transfer of information between HCPs can also be improved by using patients’ electronical medical record (EMR) to register patients’ health literacy level. To avoid negative connotation, as mentioned by a HCP in our study, this registration in the EMR always have to be with permission of the patient and needs to be carefully formulated (for example, ‘this patient requires B1-level information’). Future research could investigate the need for and most appropriate way to register patients’ health literacy level, according to both patients and providers.

We also found that, according to HCPs, most patients are not aware of the existence of the palliative team. Some of the HCPs suggested to offer a standard intake by the palliative team to every patient in the palliative phase of their disease, so the patient knows who belongs to the palliative team and knows where to find them if they need support. Another barrier for HPCs was the difficulty in initiating the conversation about the approaching end-of-life or visiting the palliative team. Flierman and colleagues also found that hospital-based professionals find it difficult to start a conversation about patients’ palliative care needs and waited for patients to state their preferences themselves [32]. Also, HCPs themselves are often not aware of the existence of a palliative team or do not refer patients to these teams. According to a previous study, hospital-based professionals feel insecure about how to define the palliative phase and also rely on interprofessional collaboration for identification although uncertainty exists about responsibilities [32]. It is also known that a timely referral of patients to a palliative care team improves their quality of life and minimizes patients’ and professionals’ distress [33].

Our e-learning includes patient education materials in plain language about ‘normal dying’, palliative sedation, euthanasia, spiritual care, reanimation and ventilation in an IC. HCPs could offer these education materials to all (LHL) patients in the palliative phase of their disease to initiate the conversation about the approaching end-of-life and the possibilities of a referral to a palliative care team. Also, palliative care teams could be more visible in general, both in and outside the hospital setting. For example by initiating team meetings for several departments and using the flow-chart from Bureau MORBidee [34] about when to initiate a conversation about palliative care.

In this study, video-recorded outpatient consultations were used to code the extent to which HCPs involved LHL patients in decision-making. Our study showed that HCPs’ application of SDM in practice, both before and after the training programme, lies between a minimal and a moderate effort. The training programme did not change the SDM level of HCPs (i.e. differences between pre- and post-measurement were not statistically significant). These findings are comparable with another study in oncological setting about palliative chemotherapy [35]. This indicates that improvement is needed, as enhanced SDM could improve patient autonomy and addressing their needs, amongst others. Therefore, continuing education and sustained effort of HCPs to improve SDM is required. SDM is a process that warrants continuous attention due to the ever-changing context in which communication takes place. For example, time pressure and the introduction of online consultations impact SDM. Future research could address the influence of context factors on SDM. However, it is also necessary to consider the role of organisational and system-level characteristics (e.g. the department and hospital itself, as well as healthcare education as a whole) to support the implementation of SDM [8]. For example, by planning ‘ booster’ team sessions about SDM (and LHL), as well as continuous monitoring of SDM skills of HCPs.

As a consequence of this evaluation, the technical issues have been solved and the e-learning has been adapted by adding: a new scenario about discussing palliative care (with a GP instead of a medical specialist or nurse) and a ‘quick route’ through the e-learning (especially for primary care providers; duration approximately 1 h). In addition, all virtual scenarios are voiced by voice actors. A strength of the e-learning is that it is freely available (with accreditation/CME points) for the participating hospitals and for medical education. Other hospitals or interested HCPs can also follow the e-learning for free, but do have to pay a small amount to get the accreditation/CME points. Moreover, more scenarios can be easily added and information updated. The e-learning is already implemented in the participating hospitals, several other hospitals and several medical curricula. By offering the e-learning as part of medical education, especially in medical schools and post-graduate medical education, future doctors and nurses become acquainted with palliative care and LHL and can practice their communication skills before they start their clinical practice. The team training has not been altered. In the future, the team training can be offered more often to hospitals as suggested by HCPs (for example, every year as a booster), by using different multidisciplinary role-play scenarios.

Our study has several limitations. First, due to job changes (and COVID-19 measures) only two individual HCPs in the participating hospitals recorded their consultations in both the pre-and post-measurement. Therefore we have compared hospitals rather than individual HCPs, assuming that HCPs did share the learned information with their (new) colleagues. Previous research also used trained hospitals to evaluate a training programme [36]. Moreover, HCPs in two hospitals could not participate in the post-measurement quantitative study (i.e. recording of their consultations), although they did want to. These HCPs had to prioritize their patients as a result of COVID-19.

Second, we did not use a controlled design. The pre-post design of this study, with a relatively small number of hospitals and consultations included, could lead to reduced external validity. Third, the extent to which HCPs apply SDM also depends on patient characteristics and the context of the consultation, as also mentioned in our previous paper [15]. We are also not aware of the specific communication and decision-making needs of LHL patients in the recorded consultation in this study. However, we know from several previous studies that most LHL patients do prefer to decide together with their HCP [31, 37]. Furthermore, the majority of the included patients in this paper were diagnosed with cancer, while the total sample (mainly in the pre-measurement) also included COPD patients. This could have influenced our findings on SDM as we found differences between the decision-making process of COPD and cancer patients [15]. However, the patient sample in this paper is comparable in the pre- and post- measurement. In addition, the recorded consultations were mainly with medical specialists (in training) and only few (specialised) nurses participated. Although, in the interviews several (specialised) nurses did participate. Overall, (specialised) nurses may have more time to communicate with patients than medical specialists, which could have influenced the application of SDM in this study [7].

## Conclusions

HCPs valued the skills they had learned during the blended training programme (e-learning and team training). HCPs specifically valued the teach-back technique, reported to have learned to use simpler wording and felt better able to recognize LHL patients. Many HCPs reported a change in communication behaviour as a consequence of the training programme. The training programme did not change the SDM level of HCPs (i.e. differences between pre- and post-measurement were not statistically significant). To improve actual SDM in practice a more sustained effort is needed, for example by using booster training sessions for HCPs and involving the organisation and system to better implement SDM. Adaptations to the e-learning have been made after this evaluation by adding a new scenario for the e-learning about discussing palliative care, amongst others. The e-learning is implemented in several hospitals and in medical education.

## Supplementary Information

**Additional file 1:** Illustrations of the e-learning and patient education materials. **Figure 1**. Overview page of the chapters of the e-learning. **Figure 2**. Virtual character from the e-learning. **Figure 3**. Virtual character from the e-learning. **Figure 4**. First pages of the patient education materials.

**Additional file 2.** Interview guide.

## Data Availability

The datasets generated and/or analysed during the current study are not publicly available due to the privacy sensitive nature of the video-recordings and interviews (see: Privacyreglement Databank Communicatie in de zorg | Nivel), but are available from the corresponding author on reasonable request.

## Abbreviations

HCPs: Healthcare providers
SDM: Shared decision-making
LHL: Limited health literacy
COPD: Chronic Obstructive Pulmonary Disease
GP: General practitioner
IC: Intensive Care
EMR: Electronical medical record
