# Supplementary material for: ‘A basic understanding’; evaluation of a blended training programme for healthcare providers in hospital-based palliative care to improve communication with patients with limited health literacy
Source: BMC Med Educ. 2022 Aug 11;22:613. doi: 10.1186/s12909-022-03685-0 (PMC9371628; doi:10.1186/s12909-022-03685-0)

**Additional file 1. Illustrations of the e-learning and the leaflets for patients**


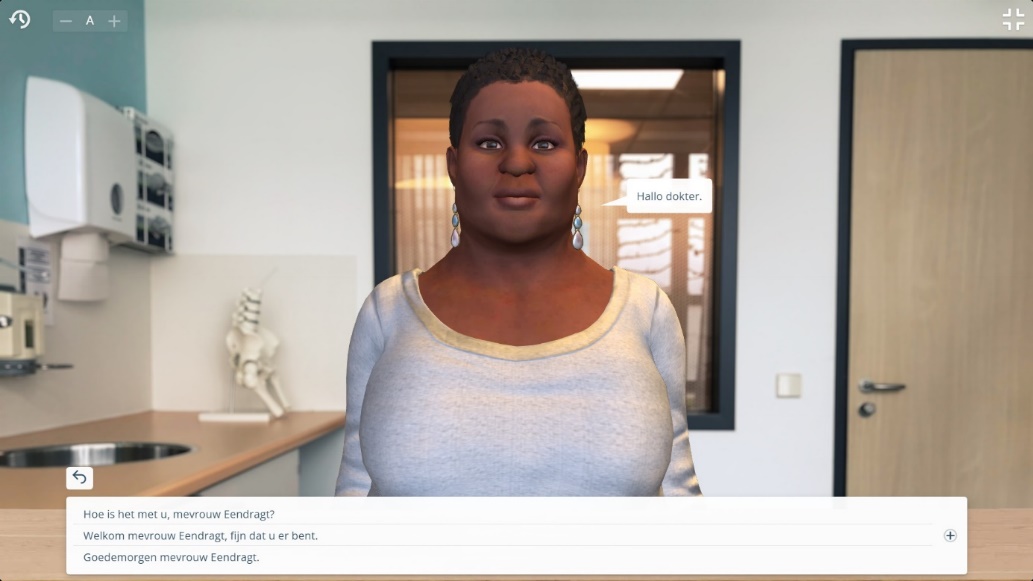
*Figure 1. Overview page of the chapters of the e-learning*


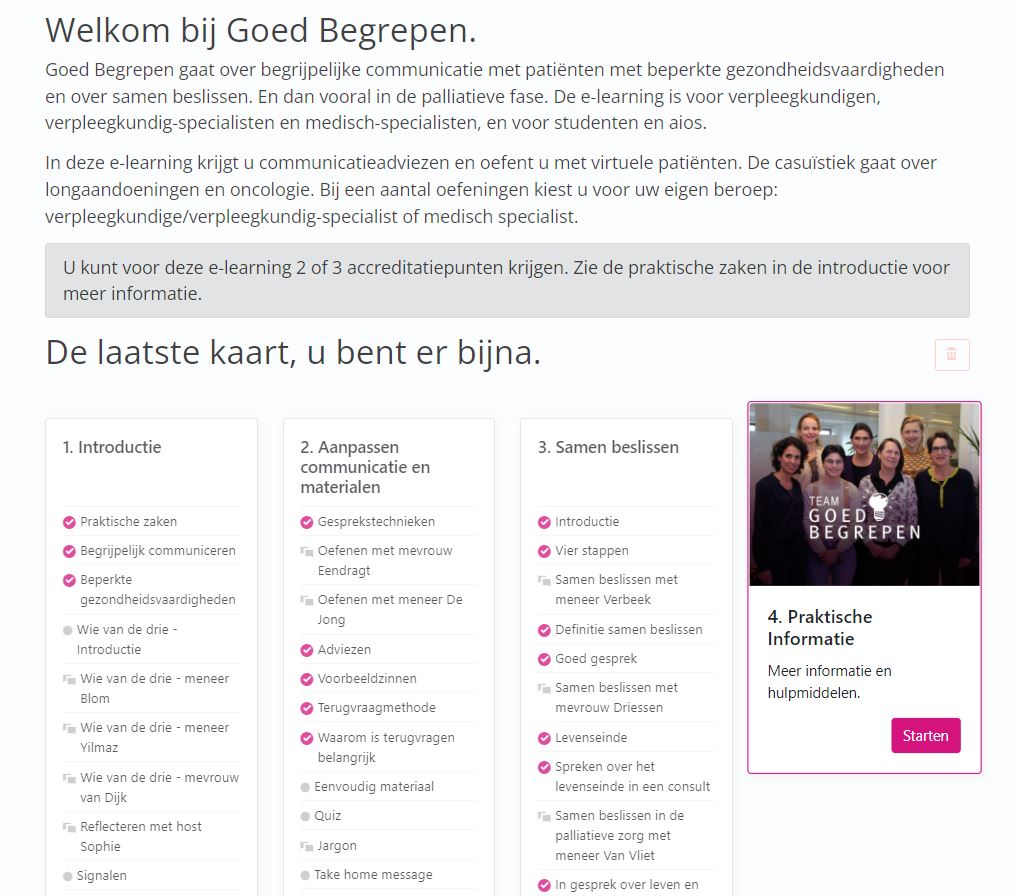


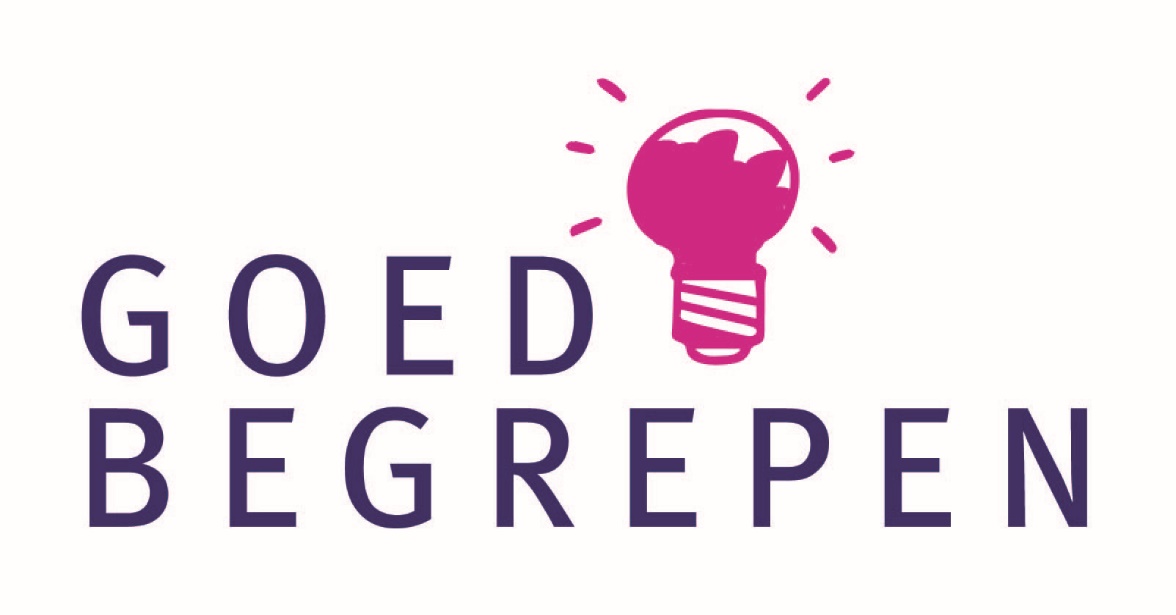


*Figure 2. Virtual character from the e-learning*

*Figure 3. Virtual character from the e-learning*


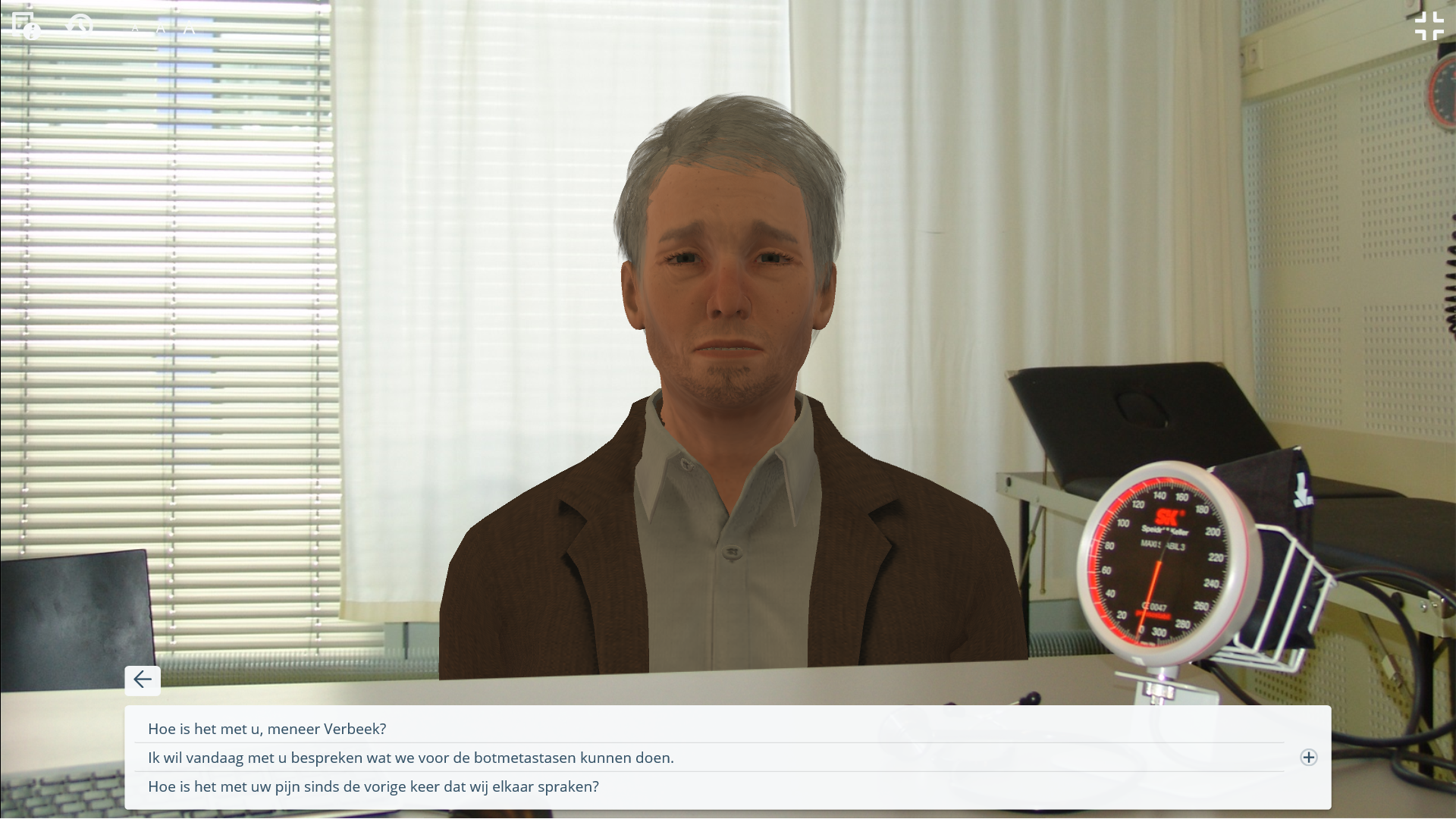


*Figure 4. First pages of the patient leaflets*


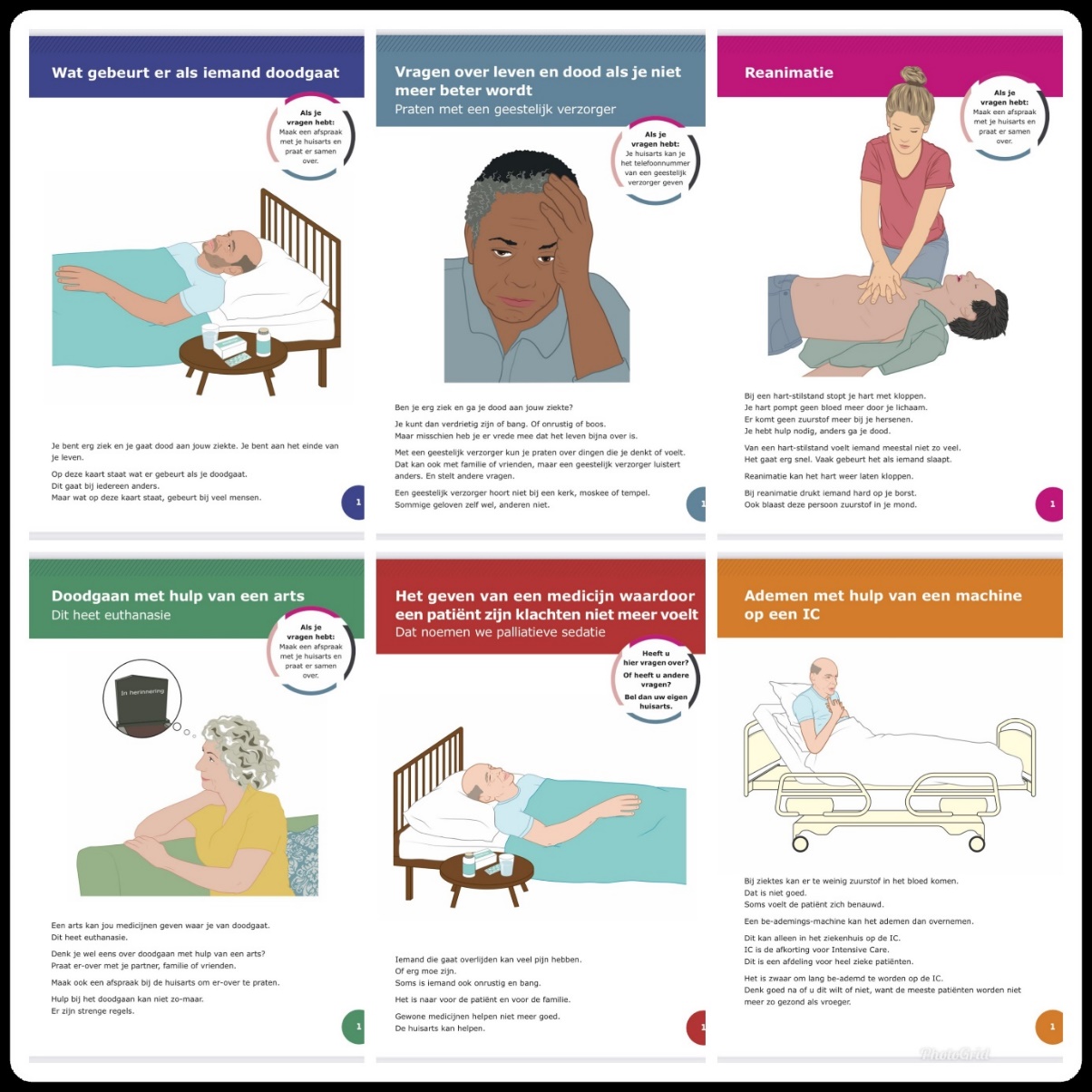

Supplement: Supplementary file 1 — Additional file 1: Illustrations of the e-learning and patient education materials. Figure 1. Overview page of the chapters of the e-learning. Figure 2. Virtual character from the e-learning. Figure 3. Virtual character from the e-learning. Figure 4. First pages of the patient education materials. [file 12909_2022_3685_MOESM1_ESM.docx]
