# Supplementary material for: ‘A basic understanding’; evaluation of a blended training programme for healthcare providers in hospital-based palliative care to improve communication with patients with limited health literacy
Source: BMC Med Educ. 2022 Aug 11;22:613. doi: 10.1186/s12909-022-03685-0 (PMC9371628; doi:10.1186/s12909-022-03685-0)
Supplement: Supplementary file 2 — Additional file 2. Interview guide. [file 12909_2022_3685_MOESM2_ESM.docx]

**Additional file 2. Interview guide**

***1. Team training***

Did you follow the team training of Pharos?

If yes:

- What did you think of the training?

- What was the most important part of the training for you?

- what was the least important part of the training for you?

- Did you adapt your communication towards patients because of the training?

- if yes, what are your experiences?

- if yes, how does this impact your conversations?

- After the training, are there any barriers in communicating with patients with limited health literacy?

- If yes, what barriers?

If no:

- What was the reason you did not follow the team training?

- Would you like to follow the team training in the future?

- if yes, are there (practical) considerations we need to keep in mind?

- if not, why not?

***2. E-learning***

*-* Did you follow the e-learning?

If yes:

- What did you think of the e-learning?

- What was the most important part of the e-learning for you?

- what was the least important part of the e-learning for you?

- Did you adapt your communication towards patients because of the e-learning?

- if yes, what are your experiences?

- if yes, how does this impact your conversations?

- After the e-learning, are there any barriers in communicating with patients with limited health literacy?

- If yes, what barriers?

- Have you used the e-learning again at a later moment?

- if yes, for what/which part?

- Do you think you will use the e-learning again in the future?

- If yes, for what? (if the participant does not answer, mention all the components of the e-learning)

- Do you have feedback for the e-learning?

If no:

- What was the reason you did not follow the e-learning?

- Would you like to follow the e-learning in the future?

- if yes, are there (practical) considerations we need to keep in mind?

- if not, why not?

***3. Communication in general (after training and e-learning)***

- What barriers do you experience in communicating with patients in general?

- What barriers do you experience in communicating with patients with limited health literacy?

- How do you take patients with LHL into account?

- How do you check if patients understand the information you provide?

- In what way do external factors play a role in your communication with patients with limited health literacy skills? (if the participant does not answer, mention for example: time, work pressure, third parties).

***4. Palliative care***

***-*** In general, how do you discuss the approaching end-of-life with patients?

- is this different for patients with limited health literacy skills?

- if yes, how?

- Who introduces the topic? (you, other HCP, patient, significant other)

- Are you familiar with the palliative team (or specific HCP for palliative care) in the hospital?

- if yes, when do you decide to contact the palliative team (or HCP) for a patient?

**Additional questions**

- Do you have other questions or suggestions about the subjects we discussed?
- Do you have comments about this interview?
